# Detroit’s Triumph

**Published:** 1900-09

**Authors:** 


### DETROIT'S TRIUMPH. THE CENTRAL WEST IN LINE.

The Detroit meeting is now a matter of history. It has been said that no country makes history faster than America. Surely everyone must be willing to accept this as true of our profession.

Less than ten years ago the A.V.M.A., then the U.S.V.M.A., planned for a meeting in Chicago, and then succeeded, through the untiring efforts of the veterinary journals and the profession in the East, of carrying there a large delegation of the members, to which was added a goodly attendance from the Central West, and then dated the success of a wider territory of Association influence. To-day a very large percentage of our membership is west of the Allegheny Mountains; then 75 per cent. was east of this dividing line. Scarcely ten years have gone by, and 1900 finds us in the region of the Great Lakes, there to hold one of the most successful meetings in the Association's history.

Think of adding in that unknown territory, so far as Association gatherings are recorded, nearly sixty new members, and losing scarcely fifteen from all causes! Surely such a growth from a membership ten years ago of less than a hundred, to nearly five hundred now, is an evidence of rapid history-making in Association progress and advancement.

